# Functional role of the additional domains in inulosucrase (IslA) from *Leuconostoc citreum *CW28

**DOI:** 10.1186/1471-2091-9-6

**Published:** 2008-01-31

**Authors:** Sandra del Moral, Clarita Olvera, Maria Elena Rodriguez, Agustin Lopez Munguia

**Affiliations:** 1 Instituto de Biotecnología, Universidad Nacional Autónoma de México. Apartado postal 510-3. C. P. 62250, Cuernavaca, Morelos, México

## Abstract

**Background:**

Inulosucrase (IslA) from *Leuconostoc citreum *CW28 belongs to a new subfamily of multidomain fructosyltransferases (FTFs), containing additional domains from glucosyltransferases. It is not known what the function of the additional domains in this subfamily is.

**Results:**

Through construction of truncated versions we demonstrate that the acquired regions are involved in anchoring IslA to the cell wall; they also confer stability to the enzyme, generating a larger structure that affects its kinetic properties and reaction specificity, particularly the hydrolysis and transglycosylase ratio. The accessibility of larger molecules such as EDTA to the catalytic domain (where a Ca^2+ ^binding site is located) is also affected as demonstrated by the requirement of 100 times higher EDTA concentrations to inactivate IslA with respect to the smallest truncated form.

**Conclusion:**

The C-terminal domain may have been acquired to anchor inulosucrase to the cell surface. Furthermore, the acquired domains in IslA interact with the catalytic core resulting in a new conformation that renders the enzyme more stable and switch the specificity from a hydrolytic to a transglycosylase mechanism. Based on these results, chimeric constructions may become a strategy to stabilize and modulate biocatalysts based on FTF activity.

## Background

Fructansucrases (E.C. 2.4.1._) or fructosyltransferases (FTFs) are enzymes that catalyze the transfer of the fructose unit from sucrose to either a growing fructan polymer chain (transglycosylase activity) or to water (hydrolytic activity). Among FTFs, levansucrases (E.C. 2.4.1.10) and inulosucrases (E.C. 2.4.1.9) are the most studied due to the physiological and industrial implications of levan and inulin, the product of their transglycosylase activity; while in levan fructose molecules are linked through β(2–6) bonds, in inulin the linkages are β(2-1), in both cases with a relative amount of branching which is dependent on the source of the enzyme.

FTFs have been reported in both Gram positive and Gram negative bacteria, but while FTFs from Gram negative bacteria have molecular weights ranging from 45 to 64 kDa [1,2] most FTFs from Gram positive bacteria present additional domains and therefore reach molecular weights as large as 170 kDa [3]. An exception is levansucrase (SacB) from *Bacillus subtilis *which has the same architecture as FTFs from Gram negative bacteria. Its structure consists of a five-bladed β-propeller single-domain fold enclosing a funnel-like central cavity, where most of the conserved residues are located including the catalytic residues Asp86 (nucleophile), Asp247 (stabilizer), and Glu342 (general acid) [PDB: 1OYG]. A detailed analysis of the structure has provided evidence of the presence of a bound metal ion, most likely Ca^2+^, which bounds to amino acids that are conserved in most of Gram-positive bacteria FTFs. In SacB, Asp339 in the sequence known as the ^339^DEIER motif makes the major contribution to Ca^2+ ^binding [4]. Ozimek et al. [5], have shown that Ca^2+ ^ions have an important structural role in levansucrase and inulosucrase from *Lactobacillus reuteri *121, suggesting that the stabilizing function of Ca^2+ ^ion is a general feature in FTFs from Gram-positive bacteria. Similarly, in Gram-negative FTFs, the calcium-binding site appears to be substituted by a disulphide bridge providing a similar fold-stabilizing role [6]. In terms of the catalytic domain, FTFs have been classified in Family 68 of Glycoside Hydrolases [7].

A subfamily of mosaic FTFs observed in *Leuconostoc spp*. containing acquired structural domains from the N and C-terminal regions of glucosyltransferases (GTFs) has recently been described [8]. Bashton and Chothia [9] have reviewed the generation of new protein functions by the combination of domains, describing how domain acquisition may confer new properties to the original enzymes such as: an increased specificity; a link between domains that have functional roles; regulate activity; combine within one chain functions that can act either independently, in concert, or in new contexts; and provide the structural framework for the evolution of entirely new functions. The authors found that in all the studied cases (45 sets of proteins), the multidomain protein has a function that is more specific or more complex than that of the one-domain protein. In the case of mosaic FTFs the consequences of this domain acquisition have not been studied. The C-terminal region in GTFs, known as the Glucan Binding Domain (GBD), has been associated in glucan polymerization, in glucan structure, in the transfer of products from the catalytic site, in cell surface localization, as well as in cell wall binding through a LPXTG motif [10-13], however, its precise role remains unknown. No specific function has been associated to the N-terminal domain, known as the variable region [3,14].

Among the mosaic FTFs, we have previously reported the characterization of inulosucrase (IslA) from *Leuconostoc citreum *CW28. IslA is a cell-associated enzyme with a molecular weight of 165 kDa [15]. As already described, this FTF presents an unusual structure: besides the variable region in the N-terminus its C-terminal domain presents 80% identity to the GBD of alternansucrase (Asr), a GTF from *L. mesenteroides *NRRL B-1355. As its catalytic domain has 36% identity to the single domain of FTF SacB, it is not probable that these additional domains may be involved in fructan specificity. However, they could be involved in other important properties of the enzyme or the products, such as stability of the enzyme, molecular weight of the polymer, reaction specificity (transglycosylation or hydrolysis), etc.

We have already demonstrated that the C-terminal domain is not essential for catalytic activity [15]. However a detailed characterization of truncated versions is required in order to explore other possible functions of these additional domains. In this work we report the biochemical characterization of inulosucrase as compared to three truncated versions: two versions with deletions in the C-terminus glucan binding domain, and one version deleted in both C- and N-terminal regions. We provide evidence demonstrating that the C-terminal region of IslA is involved in anchoring the enzyme to the cell wall; in addition, besides conferring stability, the C-terminal domain modifies the accessibility to the active site, affecting its catalytic properties. This is also demonstrated by the fact that 100 times lower EDTA concentrations are required to eliminate Ca^2+ ^ions from the catalytic domain when the C-terminal domain is removed.

## Results and Discussion

### Construction and expression of IslA truncated mutants

**Figure 1:**
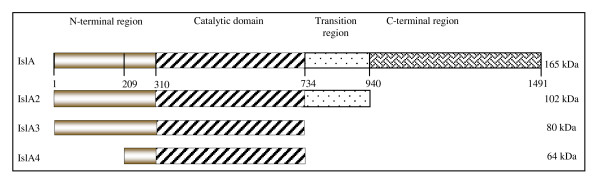
IslA truncated constructions. IslA: complete enzyme; IslA2: deletion of the C-terminal domain; IslA3: deletion of the transition and the C-terminal regions; IslA4: deletion of N/C-terminal region.

The functional role of the C- and N-terminal domains was studied through the analysis of the biochemical properties of three deleted versions of inulosucrase from *L. citreum *(IslA) described in Figure 1. IslA2 consists of a 102 kDa fragment obtained after deletion of 551 *amino *acid from the C-terminus: the deleted region is homologous to the C-terminal region of alternansucrase (Asr) from *L. mesenteroides *NRRL B-1355. The second construct is a 80 kDa variant designated as IslA3 and obtained from IslA2 but deleting also the transition region located between the C-terminal region and the catalytic domain. Finally, IslA4 is a 64 kDa fragment of IslA constituted only by the catalytic domain, after total elimination of the C-terminal region and an almost total deletion of the variable region in the N-terminus (the first 209 of 309 aa). In all cases 16 kDa corresponding to thioredoxin fused to the N-terminal regions should be included.

All proteins were produced under the control of the induced arabinose promoter in *E. coli*, resulting in active enzymes able to produce polymer. We have already demonstrated that these regions are not essential for the catalytic activity [15], as has also been demonstrated for C-terminus truncated versions of inulosucrase from *L. reuteri *[16] and for Asr from *L. mesenteroides *NRRL B-1355 [17], which retain their catalytic activity upon modification. The truncated versions are also less stable than the native enzyme. However, other consequences besides the lost of stability may result from domain acquisitions, such as changes in kinetic properties or reaction specificity.

### IslA anchors to Leuconostoc citreum cells

IslA, as well as several other FTFs and GTFs is cell associated. In some FTFs it has been demonstrated the C-terminal region is responsible for anchoring the enzyme to the cell by means of the LPXTG motif [3]. The cell associated FTF from *Streptococcus salivarius *which is devoid of motif LPXTG is released from the cells on exposure to sucrose. Through deletions within the C terminus of this enzyme, Rathsam and Jacques [18], implicated both the hydrophobic and the PGST-rich wall-associated domains in stabilizing the enzyme on the cell surface. In IslA, neither the LPXTG motif, nor the PGST motif is present. However, a blast analysis revealed a 26% identity of its C-terminal region to the cell wall binding region of amidase (Ami) from *Lysteria monocytogenes*. Ami contains 8 modules of repeat sequences designated as GW that serve to anchor the protein to lipotheicoic acids of the cell wall [19].

**Figure 2:**
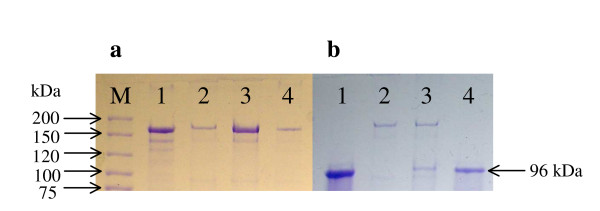
Binding assays of native IslA and the truncated form IslA3 with non induced *L. citreum *CW28 cells. Molecular weight control (M). Gel **(a) **deals with native IslA (line 1) while gel **(b) **with IslA3 (line 1). In both gels line 2 refers to the non induced *L. citreum *CW28 cells; line 3 refers to the washed non induced cells after contact with the protein; and line 4 refers to the protein solution after contact with non induced cells.

In order to determinate if the acquired C-terminal domain of IslA could be involved in cell wall anchoring, cells of *L. citreum *were produced in the absence of sucrose to avoid IslA induction. These cells were later contacted with native IslA and IslA3 (the last one is an IslA form deprived of the C-terminal region). After intensive washing, IslA was strongly retained and active in the cell surface, as demonstrated by successive activity assays and gel electrophoresis (Fig. 2a), while IslA3 was not retained (Fig. 2b), demonstrating that the C-terminus of IslA is involved in cell attachment.

We have already demonstrated the role of the C-terminal domain in DsrP, a GTF from *L. mesenteroides *IBT-PQ. In particular, the homology of DsrP and DsrE (a dextransucrase from *L. mesenteroides *NRRL B-1299) with the binding region (CW repeats) of autolysin (LytA) of *Streptococcus pneumoniae *and ToxA from *Clostridium difficile *lead us to conduct experiments that demonstrated the anchoring role of this domain in DsrP, as well as in DsrE [20]. We also conducted similar experiments with partially purified Asr from *L. mesenteroides *NRRL B-1355, which is able to bind to non induced *L. mesenteroides *NRRL B-1355 (results not shown). Asr is also cell associated and binds to the cells both in the presence or absence of its polymer [21]. Asr C-terminal region contains only a single well defined A repeat: nevertheless, this enzyme has its own seven distinctive repeat elements of nine amino acids in this region [22]. Therefore, the C-terminal region acquisition of FTF may have been a mechanism to display and anchor the enzyme in the cell surface in order to produce a biofilm a common property of these microorganisms. Several structures are displayed in *Leuconostoc spp*. FTFs and GTFs to locate them in the cell surface as can be summarized in Table 1. However the actual mechanism of protein-cell interaction is still unknown. Further experiments are required to clarify this mechanism.

**Table 1:** Proposed cell wall association motif in the C-terminal region of several of glycosyltransferases.

**Enzyme**	**Microorganism**	**motif**	**Accession number**	**Reference**
GTF-I	*S. downei*	CW repeats	P11001	[41]
GTF-B	*S. mutans*	CW repeats	AAA88588	[42]
Dsr-E	*L. mesenteroides*	CW repeats	CAD22883	[20]
Dsr-P	*L.mesenteroides *IBT-PQ	CW repeats	AAS79426	[20]
Asr	*L.mesenteroides *NRRL B-1355	GW repeats	CAB65910	This work
IslA	*L. citreum *CW 28	GW repeats	AAO25086	This work
Inu	*L. reuteri *121	LPXTG motif	AAN05575	[13]
Lev	*L. reuteri *121	LPXTG motif	AAO14618	[13]
FTF	*S. salivarius*	PGST motif	AAN87104	[43]

### Characterization of IslA and its truncated versions

#### Biochemical characterization

In order to determinate the effect of the N/C-terminal region deletion on the biochemical properties of inulosucrase, the influence of pH and temperature on the activity of the three truncated versions was studied. Although no changes were observed in the optimal pH for activity in the complete and truncated versions (pH 6.5), the pH-activity profile became sharper in the deleted forms (results not shown), most probably due to the lower stability of the truncated versions. As far as temperature is concerned, no major modifications were observed for IslA2 which retained the optimum temperature of 35°C of IslA activity; however the optimum temperature for activity decreased to 30°C when the transition region was eliminated. A deeper analysis of the truncated IslA forms stability was carried out at 35°C where it was found that the half-life of the truncated versions decrease when the acquired domains are removed (Table 2). These results, together with those reported by Olivares-Illana et al. [15], corroborate that the N/C-terminus contribute to stabilize the catalytic domain. This is also the case of DsrS, where the truncated version is more susceptible to thermal denaturation [23]. However, the deletion of the C-terminal region does not always result in a lost of stability, as in the case of Asr from *L. mesenteroides *B-1355 where deletions of the N/C-terminus (with high identity to the C-terminal domain of IslA) do not affect the thermal stability of the truncated forms [17].

**Table 2:** Biochemical and kinetic properties of inulosucrase (IslA) from *L. citreum *CW28 and truncated versions.

**Truncated version**	**Optimum T (°C)**	**Half life @ 35°C (min)**	**Hydrolysis/transglycosylase ratio (%)**^a^	**Km**^T ^**(mM)**	**kcat (s^-1^)**	**kcat/Km (mM**^-1 ^**s**^-1^**)**
IslA	35	420	40/60	38	25	0.65
IslA2	35	407	38/62	24	28.19	1.17
IslA3	30	346	61/39	nd	nd	nd
IslA4	30	128	70/30	142	105	0.73

^a ^Measured after depletion of 293 mM sucrose. Km^T ^(total Km) was determined from sucrose consumption rate.

IslA3 did not follow a Michaelis Menten kinetic behavior

The polymer structure and the molecular weight of the polysaccharides produced by the truncated versions were analyzed by means of ^13^C NMR: the spectra of the polymer synthesized by the IslA mutants was identical to the one obtained from the complete IslA protein, equivalent to a fructose polymer linked through β(2-1) bonds and identified as inulin (data not shown). The protein deletions have also no influence in the polymer molecular weight distribution as observed by gel permeation HPLC: all polymers have a molecular weight distribution in the range of 90 000 to 4 400 000 Da, similar to the polymer produced by IslA. It is therefore possible to conclude that, although a detailed analysis of the polymer size is difficult to perform, there are no major differences in product specificity of the mosaic FTFs and the deleted forms including, IslA4 which could be considered equivalent to single domain FTFs. This phenomenon has also been observed in Asr, where the deletion of the C-terminal region did not affect the properties of the product [17]. Similar consequences were observed with C-terminus truncated versions of inulosucrase from *L. reuteri *[24] and GTF-I from *Streptococcus downei *[25]. Nevertheless it is not possible to generalize this behaviour as when the C-terminal region was deleted from GTF-I from *S. mutans*, the resulting enzyme lost completely its capacity to synthesize the polymer, retaining only sucrase activity [26].

#### Kinetic properties

Total Km and kcat values were determined for IslA as well as for the truncated versions from initial sucrose consumption rates (Table 2). In this case, all forms exhibit Michaelis-Menten type kinetics, with the exemption of IslA3 which was best described by the Hill equation. The Hill equation has also been applied to describe the kinetic behavior of inulosucrase and levansucrase from *L. reuteri *at 50°C [16] which do not exhibit a saturating behavior. Although there is no net modification in the catalytic efficiency of the IslA forms as measured by the kcat/Km ratio, some interesting observations result from the analysis of the individual parameters. Even when it is difficult to define a trend in terms of the apparent total Km value, it is possible to observe that the smallest IslA versions, lost sucrose affinity as concluded from a one order of magnitude increase in its total Km value. An interesting feature is that there is also a 2–4 fold increase in its total kcat value, as if partial elimination of the structure would result in a facilitated access of the substrates, particularly the catalytic water to the active site. Changes in sucrose affinity have also been reported in the truncated GTF-A from *L. reuteri *[27] which increased its Km with respect to the native enzyme. It is interesting to point out that the total Km value of IslA and IslA2 is similar to the value reported for most FTFs including both single domain enzymes such as levansucrases from *L. reuteri *(21 ± 4 mM) [16], *A. diazotrophicus *(11.8 mM ± 1.4) [28], *L. sanfranciscensis *(13.1 mM) [11] or multidomain FTFs such as *L. mesenteroides *NRRL B-512F (LevS) (36.7 ± 5.4 mM) [29] and *L. mesenteroides *ATCC 1359 (LevC) (27.3 mM) [8]. Interestingly, the lost of affinity, makes it equivalent, in terms of the total Km value, to single domain levansucrases from Gram negative bacteria, such as *Z. mobilis *[30] and *P. syringae *[1] (160 and 122 mM respectively).

We have already demonstrated that IslA, as most FTFs and GTFs, have a transglycosylase activity which is a function, among others parameters, of sucrose concentration [31]. When this property was studied for the truncated versions in a wide substrate concentration range (up to 0.87 M sucrose) it was found that, as expected, the higher the sucrose concentration, the higher the transglycosylase activity. In spite of this result, observed for all IslA forms, a higher hydrolytic activity was found when the transition region was eliminated, as shown in Table 2. These results suggest that in the chimeric construction, the acquired domains, in particular the transition region, may interact with the catalytic core, turning the enzyme less hydrolytic, probably due to the conformation of a larger path for the accessibility of the catalytic water molecules to the active site. In any case, the higher the hydrolytic activity of the IslA form, the higher its kcat value (Table 2), as a consequence of a preferential transfer of the fructosyl residue to water than to the polymer acceptor. In the same context, other factors reducing the hydrolysis in favor of the transglycosylase activity in FTFs include the use of organic solvents [32,33] or the immobilization of the enzyme [34]. It is interesting to observe that in these last cases (high substrate concentration, use organic of solvents or enzyme immobilization) the common feature is the reduction of water activity (a_w_) in the vicinity of the active site.

#### Effect of the additional regions on calcium diffusion

A putative calcium binding site coordinated by Asp339 of the ^339^DEIER motif, where the Glu342 catalytic residue is also found, has been determined in SacB crystallographic structure [4]. The authors speculate that in the absence of Ca^2+ ^ions the ^349^DEIER loop acquire a conformation less favorable for catalysis.

**Figure 3:**
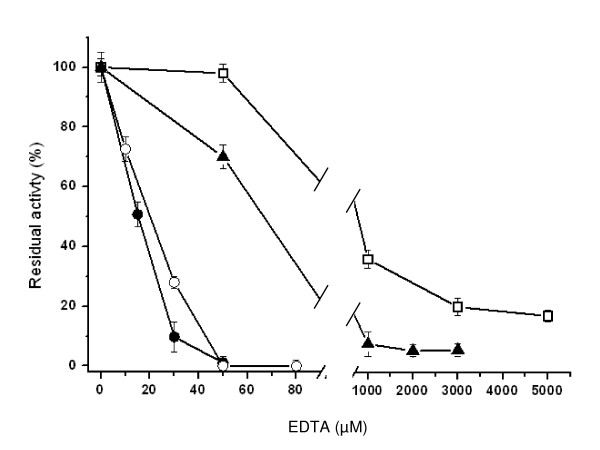
Effect of EDTA on native IslA and truncated forms activity. IslA4 (black circles), IslA3 (open circles), IslA2 (black triangles), IslA (open squares). Activity measurements are made after 5 min of incubation with EDTA for the IslA4 and IslA3 forms, and after 180 min for native IslA and IslA2.

Considering this structural feature, the Ca^2+ ^ion effect on IslA4 activity was evaluated. It was found that the activity is lost when Ca^2+ ^ions are depleted using EDTA. However, when this effect was analyzed in detail, the rate of Ca^2+ ^ions depletion was found to be structure-dependent, as measured by the rate of activity loss and the higher EDTA concentrations required to remove Ca^2+ ^ions in short times. These results are summarized in Figure 3, where it may be observed that while 50 μM of EDTA and 5 minutes of incubation are enough to deplete IslA4 and IslA3 of Ca^2+ ^ions and activity, 1000 μM and 180 min are required to achieve the same effect with IslA2. Interestingly, after 40 h incubation of IslA with 5000 μM EDTA, that is 100 fold more EDTA that the concentration used with IslA4 and IslA3, the enzyme still retains 20% of the original activity. This is probably due to the fact that the three domain IslA require Ca^2+ ^ions to optimize the activity, but is not essential, a property that would be inherent to the mosaic structure. In any case, the interaction of the additional domains, particularly the transition region, with the catalytic core put forward to describe the hydrolysis/transglycosylase reaction ratio variations, could also explain the difficulties to remove Ca^2+ ^ions (Fig. 3) a consequence of a less exposed DEIER motif.

#### Structural changes influenced by Ca^2+ ^ions on the truncated forms

**Figure 4:**
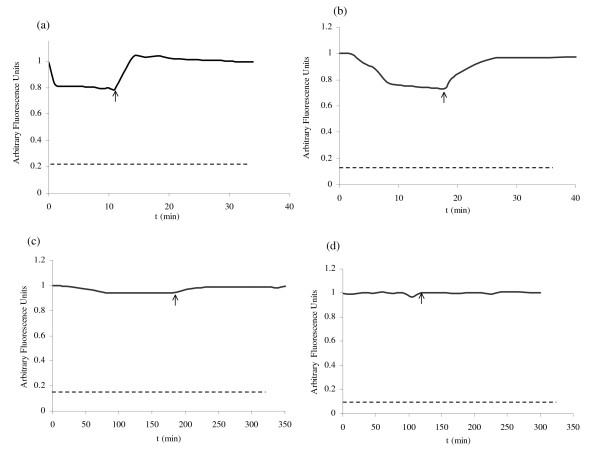
Structure modifications of IslA and truncated forms mediated by EDTA and Ca^2+ ^ions measured by intrinsic fluorescence (excitation wavelength = 280 nm, emission wavelength = 348 nm) (a) IslA4, 50 μM EDTA and 500 μM Ca^2+ ^ions; (b) IslA3, 50 μM EDTA and 500 μM Ca^2+ ^ions; (c) IslA2 1000 μM EDTA and 2000 μM Ca^2+ ^ions and (d) IslA 5000 μM EDTA and 7000 μM Ca^2+ ^ions. EDTA is added after the first fluorescence measurement; time of Ca^2+ ^ions addition is indicated by an arrow. For each case, the fluorescence of the heat denaturated protein is shown as a broken line.

**Figure 5:**
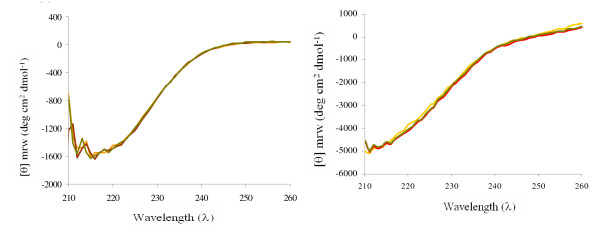
Circular Dichroism spectra of native IslA and IslA4 mediated by EDTA and Ca^2+ ^ions. (a) IslA4: control (yellow line), IslA4 after incubation for 15 min with 0.5 mM EDTA (brown line); IslA4 after incubation for 15 min of the previous sample after restoring Ca^2+ ^ions (1 mM) (green line). (b) IslA: control (yellow line), IslA after incubation for 180 min with 0.5 mM EDTA (brown line); IslA after incubation for 180 min of the previous sample after restoring Ca^2+ ^ions (8 mM) (green line).

In order to perform a detailed analysis of the role of the additional domains in inulosucrase stability, the influence of Ca^2+ ^ions in the conformational structure of the native and truncated versions was studied through the measurement of intrinsic fluorescence in presence of EDTA and Ca^2+ ^ions. For this purpose the fluorescence intensity was followed after EDTA addition; when a constant intensity was reached, Ca^2+ ^ions were restored. The time scale as well as EDTA and Ca^2+ ^ions concentration was dependent on the IslA form studied. It may be observed that IslA4 (Fig. 4a) and IslA3 (Fig. 4b) undergo slight local structural modifications when Ca^2+ ^ions are depleted by addition of 50 μM EDTA, as deduced from the fluorescence intensity change. However, a rapid recover occurs when 500 μM CaCl_2 _are restored. In the case of IslA4 and IslA3 a complete unfolding is not reached, contrary to the observations made in SacB [35] by Petit-Glatron *et al*, who found that SacB suffers a complete unfolding which is reverted when Ca^2+ ^ions are restored. On the other hand, Circular Dichroism (CD) experiments confirmed that the changes observed in fluorescence during Ca^2+ ^depletion did not result in any modification of the IslA4 secondary structure, as concluded from the CD spectrum shown in Figure 5a.

Surprisingly, no significative changes in fluorescence intensity in the absence of Ca^2+ ^ions were observed for IslA2, even in the presence of 1000 μM EDTA that inactivates the enzyme (Fig. 4c). Similarly, the fluorescence intensity measurements of IslA in the presence of 5000 μM EDTA during 180 min, imply that no modifications take place, even when the enzyme retains only 20% of original activity. The CD experiments performed on IslA in the absence of Ca^2+ ^ions demonstrated no changes in secondary structure strengthening the hypothesis that the additional domains confer rigidity to the enzyme, generating a more stable form even in the absence of Ca^2+ ^ions. In summary, the smallest versions of IslA: IslA3 and IslA4, loose activity in the absence of Ca^2+ ^ions (Fig. 3) with slight modifications in their tertiary structure (Fig 4); these changes are reverted when Ca^2+ ^ions are restored. Throughout this process, the secondary structure of IslA4 is conserved. In contrast, IslA and IslA2 retain around 20% and 10% of the original activity respectively, even in presence of high EDTA concentrations without alterations in its tertiary structure, indicating that the transition and the C-terminal regions confer stability to the protein.

## Conclusion

Through binding assays, we demonstrated that the C-terminal domain in inulosucrase IslA serves to anchor the enzyme to the cell surface. The difficulties found to remove Ca^2+ ^ions as the structure becomes more complex, from IslA4 to IslA, together with the greater sucrose affinity (smaller Km) and the higher thermostability, allow also us to conclude that the acquired domains in IslA interact with the catalytic core resulting in a new conformation that renders the enzyme more stable and generates a switch in specificity from an hydrolytic to a transglycosylase mechanism. Actually, this strategy in nature has been recently observed elsewhere in a completely different enzyme structure and activity. Trehalose synthase has been reported both as a single domain enzyme in *Deinococcus radiodurans, Pseudomonas sp, Pimelobacter sp*. [36,37], and as mosaic proteins with α-amylase regions acquired in the C-terminal domain in *Thermus thermophilus *[38]. Wang et al. [38] through deletion of the acquired regions demonstrated also that the single domain enzyme is not only less stable but hydrolyzes more trehalose.

## Methods

### Cloning and expression of truncated versions

In a previous work, truncated versions were constructed in order to explore if the C-terminal domain was essential for activity [15]. In this work, the same truncated versions were fused to a His tag and expressed under the *ara *promoter in order to produce and purify enough protein for characterization. Each gene fragment was amplified from *islA *cloned in plasmid pCR-TOPO [15] using the corresponding primers: IslA2 *IS2reverso *(CTAATTTAAATCGCGTGAAAAGCTAATGGC) and *SPdirevecto *(ACCATGGACG TGAATCAACCACTTTTAGCG); IslA3 *ISE3rvEco *(ATC CTC AGA ATT CAA TGC TAA TAA CTC AAC) and *SP directo; *IslA4 *BproNae *(GAA ATG ACT AGT GTG CCG GCG CTT ATA TC) and *ISE3rvEco*. The amplification products were cloned into the pBAD/Thio TOPO expression vector (Invitrogen, Calsbad, CA). *E. coli *strain TOP10 was used to transform the constructed plasmids and to express the truncated IslA truncated versions. Overnight cultures of the transformed strains, carried out at 37°C in 50 ml Luria-Bertani medium supplemented with 100 μg/ml ampicillin, were used as inoculum of 950 ml of the same medium and grown until an 0.6 OD_600 nm _was reached. At this time, expression of the recombinant proteins was induced by addition of 0.02% (w/v) L-arabinose for IslA2 and 0.2% (w/v) for IslA3 and IslA4 and the temperature reduced to 23°C. Cells were harvested at 1.8 OD_600 nm_.

### Preparation of E. coli cell extracts and purification of IslA and truncated versions

*E.coli *cells were harvested by centrifugation (10 min, 4°C, 4600 g). The resulting pellet was washed twice with 50 mM pH 6.5 phosphate buffer. Afterwards, cells were suspended in 5 ml of the same buffer and broken at 900 psi in a French press. Cell debris was removed by centrifugation for 30 min at 4°C at 10000 g and the supernatant assayed for activity.

The enzymatic forms were purified by affinity chromatography through their His tags. A bed volume of 600 μl of Ni-nitroacetic acid (Ni-NTA) resin (Qiagen) was used to bind protein from 5 ml of cell extract. The resin was equilibrated with 3 ml of binding buffer (NaH_2_PO_4 _50 mM, NaCl 300 mM, imidazole 10 mM) pH 7.5 for IslA2 and pH 7 for IslA3 and IslA4. The cell extract was diluted 1:1 with buffer binding and incubated for 1 h at 4°C with the equilibrated resin, followed by washing with 7 ml of the same buffer containing 30 mM imidazole. Finally the recombinant protein(s) were eluted with 2 ml of elution buffer (50 mM NaH_2_PO_4_, 300 mM NaCl, 250 mM imidazole). The proteins were dialized against 50 mM pH 6.5 phosphate buffer and 1 mM CaCl_2_. The cell-associated IslA was extracted from *L. citreum *CW28 cells with 8 M urea at 25°C for 1 h with occasional gentle shaking as already described [31]. The extract was then dialyzed against 50 mM pH 6.5 phosphate buffer, after centrifugation. With this procedure, IslA was obtained in a highly purified form. The purity of the enzyme and truncated forms was verified by SDS-PAGE 8%.

### FTF activity assay

Initial reaction rates of IslA and truncated versions were measured at 30°C in 50 mM pH 6.5 phosphate buffer in the presence of 293 mM sucrose and 1 mM CaCl_2_. The activity was measured by following of the reducing power released from sucrose by the 3,5-dinitrosalicylic acid method (DNS). One activity unit (U) is defined as the amount of enzyme that produces 1 μmol of glucose per minute. Specific activity is reported as U/mg of protein. The protein concentration was determined by the Bradford method [39], using the Bio-Rad reagent and BSA as standard. In a more specific assay, glucose and fructose were analyzed by HPLC in a Waters instrument equipped with a refraction index detector (Waters 410) and using a high performance carbohydrate cartridge (Waters) at 35°C and acetonitrile:water 75:25 as eluent at a flow rate of 1.4 mL/min.

### Biochemical and enzymatic characterization of IslA and truncated versions

IslA and truncated versions activity was assayed in the 20 to 40°C temperature range in 50 mM pH 6.5 phosphate buffer and 1 mM CaCl_2_, while the effect of pH was determined in the 5.0 to 8.0 range in the same buffer. All the experiments were performed in triplicates.

Kinetics properties were studied through initial rate of reaction measurements carried out at pH 6.5 and 30°C in sucrose solutions ranging from 14.6 to 584.8 mM and containing 1 mM of CaCl_2. _Samples of 50 μl were withdrawn after addition of the enzyme at 3 min time intervals and poured into 50 μl of DNS solution to stop the reaction and perform the reducing power assay. The data was processed using the Hills or the Michaelis-Menten equations. The transglycosylase and hydrolase activities of IslA and truncated versions were determined from the glucose and fructose concentrations measured by HPLC.

### Ca^2+ ^ions binding

Ca^2+ ^ions were depleted by addition of EDTA in amounts that were found dependent on the protein structure, as described in the results section. Accordingly, different concentrations of Ca^2+ ^ions were used to restore the activity.

### Fructan characterization

Fructan was produced with all the enzymes forms at 30°C in 50 mM pH 6.5 phosphate buffer containing 100 g/L sucrose and 1 mM CaCl_2_. The polymer was precipitated with two volumes of ethanol, dialyzed against water, lyophilized and analyzed by ^13^C NMR. Inulin MW was analyzed by gel permeation chromatography in a Waters 600E HPLC system controller (Waters Corp. Milford, MA) equipped with a refractive index detector (Waters 410), using a serial set of Ultrahydrogel columns (UG 500 and linear) at 35°C with water as mobile phase at 0.9 mL/min.

### Secondary and tertiary structure determination

Tertiary structure of the truncated forms was examined by Trp fluorescence assays on a Perkin Elmer LS-55 spectrofluorimeter. The proteins were purified and filtered and solutions prepared containing 0.018 mg/mL of IslA4; 0.02 mg/mL of IslA3 and IslA2; and 0.01 mg/mL of IslA. The proteins were excited at 280 nm and the fluorescence emission measured at 348 nm at 30°C. The secondary structure of the truncated versions was determined by Circular Dichroism (CD) from solutions containing 5.04 μM of IslA4 and IslA3 and 1.7 μM of IslA in 50 mM pH 6.5 phosphate buffer (CaCl_2 _and/or EDTA were added according to the case). The solutions were placed in quartz cuvettes of 2 mm path length and CD spectra in the far UV region (190–250 nm) recorded on a Jasco J-715 spectropolarimeter at 25°C. All the spectra shown is the average of 3 scans recorded at a scanning rate of 20 nm/min. Spectra were corrected by subtracting appropriate buffer blanks and smoothed by noise reduction.

### Cell wall anchoring assay

In order to demonstrate the role of the C-terminal domain in binding to *L. citreum *CW28 cells, we first produced non induced *L. citreum *CW28 cells in LM culture supplemented with glucose 2% (w/v) instead of sucrose, harvested at 5 OD_600 nm _by centrifugation and washed twice with 50 mM pH 6.5 phosphate buffer. Cell protein was measured by the Lowry method [40]. In a second step, 0.5 mg/ml of the purified proteins IslA and IslA3 (with and without the C-terminal domain respectively but retaining enzymatic activity) were incubated with 2.5 mg/ml final concentration of non induced *L. citreum *CW28 cells for 12 h at 4°C with gentle shaking. Afterwards, cells were separated by centrifugation at 4°C, 12000 g. The pellet was washed three times with 50 mM pH 6.5 phosphate buffer, and for both, supernatant and the pellet, the FTF activity was determined and SDS-PAGE 8% gels were performed.

## Authors' contributions

SMV generated, expressed and purified the truncated versions and carried out the structural characterization, the binding assays, and drafted the manuscript; MERA realized the biochemical characterization of truncated versions; COC participated in experimental design and coordination. ALM conceived and supervised the project and manuscript writing. All authors read and approved the final manuscript.
